# Endothelial function and urine albumin levels among asymptomatic Mexican-Americans and non-Hispanic whites

**DOI:** 10.1186/1476-7120-6-43

**Published:** 2008-08-27

**Authors:** Julius M Gardin, Zuhair Allebban, Nathan D Wong, Sharon K Sklar, Renee L Bess, M Anne Spence, Harrihar A Pershadsingh

**Affiliations:** 1 Division of Cardiology, Department of Medicine, St. John Hospital & Medical Center, Detroit, MI, 48236, USA; 2 University of California-Irvine, Irvine, CA, 92697, USA; 3 Kern Medical Center, Bakersfield, CA, 93305, USA; 4 Department of Internal Medicine, Hackensack University Medical Center, and Touro University College of Medicine, 30 Prospect Avenue, Hackensack, New Jersey, 07601, USA

## Abstract

**Background-:**

Mexican-Americans (MA) exhibit increases in various cardiovascular disease (CVD) risk factors compared to non-Hispanic Whites (NHW), yet are reported to have lower CVD mortality rates. Our aim was to help explain this apparent paradox by evaluating endothelial function and urine albumin levels in MA and NHW.

**Methods-:**

One hundred-five MA and 100 NHW adults were studied by brachial artery flow-mediated dilatation (FMD), blood and urine tests. Participants were studied by ultrasound-determined brachial artery flow-mediated dilatation (FMD), blood and urine tests, at a single visit.

**Results-:**

Despite higher BMI and triglycerides in MA, MA demonstrated higher FMD than did NHW (9.1 ± 7.3% vs. 7.1 ± 6.3%, p < 0.04). Among MA, urinary albumin was consistently lower in participants with FMD ≥ 7% FMD versus < 7% FMD (p < 0.006). In multivariate analyses in MA men, urinary albumin was inversely related to FMD (r = -0.26, p < 0.05), as were BMI and systolic blood pressure. In MA women, urinary albumin:creatinine ratio was an independent inverse predictor of FMD (p < 0.05 ).

**Conclusion-:**

To our knowledge, this is the first study to analyze, in asymptomatic adults, the relation of MA and NHW ethnicity to FMD and urine albumin levels. The findings confirm ethnic differences in these important subclinical CVD measures.

## Background

Mexican-Americans (MA) are known to exhibit increased prevalence of various cardiovascular disease (CVD) risk factors – e.g., obesity, hyperlipidemia and diabetes mellitus – compared to non-Hispanic whites (NHW), yet have been reported to have lower CVD case fatality rates than NHW [1-3]. Although endothelial dysfunction (ED) is known to be an early marker of vascular disease-e.g., atherosclerosis-there is a lack of data examining ethnic differences in ED between *asymptomatic *MA and NHW.

The Corpus Christi Heart Project investigators suggested that their finding of a greater hospitalized myocardial infarction rate – in the face of reported lower coronary heart disease (CHD) fatality rates among MA in vital statistics studies – is likely due to a misclassification of cause of death and/or ethnicity on death certificates [4]. However, another possible explanation may be differences in degree of subclinical CVD [5].

Flow-mediated dilatation (FMD) of the brachial artery measured using ultrasound has been shown to be an early predictor of the risk of developing CVD [2]. Microalbuminuria, also hypothesized to be related to vascular endothelial function, has previously been described as the earliest marker for the development of CHD in Type I diabetes mellitus [6]. The aim of this pilot study was to obtain FMD and urinary albumin data in MA and NHW that might help explain the apparent paradox in MA. The hypotheses tested were: 1) There are significant differences in FMD between MA and NHW; these differences relate to ethnic differences in the levels of traditional CHD risk factors; and 2) FMD is associated with subclinical disease another urinary albumin measure, in one or more of these ethnic/gender subgroups.

## Methods

### Subjects

The study was conducted among asymptomatic community-based adult volunteers. To qualify for inclusion, subjects had to be classified as Mexican-American (MA) according to at least one of the following criteria used in the San Antonio Heart Study [7]: 1) Father's surname and mother's maiden name are both Spanish, and both parents were born in Mexico; and/or 2) Only one parent has a Spanish surname, but three of four grandparents have Mexican origins.

We studied 105 adult MA (42 men and 63 women, age 46 ± 14 yrs) and 100 NHW (59 men and 41 women, age 50 ± 11 yrs) volunteers using blood tests, transthoracic echocardiography (echo), and brachial arterial flow-mediated dilatation (FMD) by ultrasound. Subjects were generally healthy: Individuals with hypertension or known CVD, or taking cardiovascular or BP medications were excluded.

### Echocardiography

This study employed the echo protocol used in both the Cardiovascular Health Study (CHS) [8] and Coronary Artery Risk Development in Young Adults (CARDIA) studies [9]. For each subject, a baseline echo was recorded using a standardized protocol. Two-dimensionally guided M-mode echo measurements of the LV and left atrium were made according to conventions of the American Society of Echocardiography. LV mass was derived from the formula described by Devereux, et al. LV mass was normalized for various body size measures, including height [10].

### Measurement of Flow-mediated Dilatation

A 7.0 or 10 MHz linear array ultrasound transducer was used to image the right brachial artery 6 cm proximal to the antecubital fossa. Scanning was performed in the longitudinal view with transmit (focal) zone set to the depth of the arterial near-wall. After recording baseline images, a right brachial artery BP cuff was inflated to 30 mmHg above systolic pressure, occluding the artery for 4 minutes. The right brachial cuff was then deflated rapidly to zero pressure, resulting in reactive hyperemia. The arterial segment imaged at baseline was continuously imaged during cuff inflation and for 3 minutes following deflation. Brachial artery diameter after reactive hyperemia was expressed as a percentage of resting diameter.

### Blood/Urine Samples

Fasting venous blood samples were obtained for analysis of serum electrolytes and creatinine; fasting total, HDL-, and LDL-cholesterol and triglycerides; and glucose. A spot urine was collected for albumin and creatinine determinations.

### Statistical Analysis

Data are presented as mean ± standard deviations or percentages. Group comparisons were performed using Student's t-test for continuous variables and X^2 ^test for categorical variables. Due to a lack of data indicating an appropriate percent cut-point for normal vs. abnormal FMD in the MA population, the reported cut-point for NHW (abnormal FMD < 7%) was examined in this study [11-13].

Multiple linear regression analyses were performed to identify risk factors simultaneously predictive of FMD. Variables were selected for entry if the probability value was < 0.10 in univariate testing of association. Modeling was done using the selected factors as independent variables and FMD as dependent variables. The full set of potential factors was considered with the forced entry method. To examine whether ethnicity (MA vs. NHW) remained significantly associated with FMD after adjusting for cardiovascular risk factors, multivariate analyses were conducted adjusting for parameters independently related to FMD – e.g., BMI, age, gender, systolic BP, LDL-cholesterol, brachial artery diameter, etc. – with and without urinary albumin. Additional multivariate analyses were performed using analysis of covariance (ANCOVA) with estimated marginal means and main effect options compared to determine the relation of urine albumin and CVD risk factors to FMD. ANCOVA models were created to assess the association between FMD and the various risk factors (and urine albumin) while adjusting for age or age and risk factors. The effect of each risk factor with a significant bivariate relationship to FMD was analyzed by ANCOVA. Models were constructed by adjusting for age (Model 1), age plus CVD risk factors (systolic BP, BMI, LDL-cholesterol, HDL-cholesterol, triglycerides, glucose, and current smoking) (Model 2), and age, CVD risk factors plus either urinary albumin (Model 3), or urine albumin:creatinine (Alb:Cr) ratio (Model 4).

## Results and Discussion

Study participant demographic, risk factor, and subclinical disease characteristics by ethnic group and gender are presented in Table 1. BMI and serum triglycerides were significantly higher in the overall MA versus the overall NHW cohort, while age, LDL-cholesterol, serum creatinine, and percent current smokers were all significantly lower in the MA than in the NHW cohort.

**Table 1:** Distribution of flow-mediated dilatation, and demographic and cardiovascular risk factors among Mexican-American (MA) and non-Hispanic white (NHW) cohorts of men and women

	**Overall**		**Men**		**Women**	
	Mean ± SD		Mean ± SD		Mean ± SD	
**Parameter**	**MA **(N = 105)	**NHW **(N = 100)	**MA **(N = 39)	**NHW **(N = 59)	**MA **(N = 66)	**NHW **(N = 41)
**Risk Factors**						
Age (yrs)	46 ± 13.5	49.6 ± 10.8*	46.3 ± 13.6	47.7 ± 11.3	45.9 ± 13.6	52.4 ± 9.5**
Body Mass Index (kg/m^2^)	30.3 + 7.1	27.8 + 4.2**	30.9 ± 6.1	28.4 ± 3.5**	29.98 ± 7.6	26.9 ± 4.95*
Triglycerides (mg/dl)	172.6 ± 115.9	124.9 ± 66.6**	184.7 ± 92	135.7 ± 71**	166 ± 126.9	109.4 ± 56.2**
LDL cholesterol (mg/dl)	110.7 ± 29.4	123.5 ± 30.4**	112.8 ± 27.7	127.5 ± 27.7**	109.5 ± 30.4	117.8 ± 33.5
HDL cholesterol (mg/dl)	53.6 + 13.9	53.7 + 12.8	45.5 + 6	48.8 + 10*	57.9 + 14.2	60.9 + 12.6
Total Cholesterol (mg/dl)	198.7 ± 35.9	203.5 ± 37.5	195.6 ± 33.1	205.5 ± 37.9	200.3 ± 37.4	200.6 + 37.2
Systolic BP (mmHg)	124.5 ± 17.4	125.2 ± 14.4	129 ± 12.9	127.4 ± 13.4	121.9 ± 19	122.1 ± 15.4
Diastolic BP (mm Hg)	72.3 ± 10.6	74.6 ± 9.8	79.1 ± 9.9	77.4 ± 9.9	68.5 ± 9	70.5 ± 8.3
Serum Glucose (mg/dl)	90.8 ± 13.4	88.7 ± 21.7*	91.7 ± 13.1	91.2 ± 26.6	90.3 ± 13.7	85 ± 10.8*
Serum Creatinine (mg/dl)	0.8 ± 0.2	0.9 ± 0.2**	0.94 ± 0.1	0.98 ± 0.1*	0.73 ± 0.1	0.8 ± 0.1**
Urinary albumin (mg/dl)	0.88 ± 0.85	1.1 ± 2.3	0.9 ± 0.9	1.3 ± 2.9	1.1 ± 1.1	0.5 ± 0.3**
Brachial Artery Baseline						
Diameter (cm)	0.389 ± 0.06	0.393 ± 0.07	0.41 ± 0.05	0.418 ± 0.04	0.322 ± 0.04	0.32 ± 0.05
Non-smokers (%)	65%	39%**	19%	27%	46%	14%**
Current smokers (%)	35%	61%**	16%	33%	19%	26%
						
**Subclinical Disease**						
Flow-mediated Dilatation %	9.1 ± 7.3	7.1 ± 6.3*	8.7 ± 6.6	6.6 ± 4.1	9.2 ± 7.4	8.9 ± 7.3

*: p < 0.05; **: p < 0.01. P values represent the comparison between MA vs NHW for overall, men and women cohort. Abbreviations: LDL: low density lipoprotein; HDL: high density lipoprotein.

In the overall cohort, MA demonstrated higher FMD compared to NHW (absolute diameter change: 0.0354 ± .02 cm vs 0.0280 ± 0.03 cm and % FMD: 9.1 ± 7.3% vs 7.1 ± 6.3%, respectively, both p < 0.04). Brachial artery baseline diameters were similar in MA and NHW (0.389 ± 0.06 and 0.393 ± 0.07 cm, respectively).

### Bivariate analyses

Bivariate analyses of the relationship between risk factor and subclinical disease variables and FMD were performed by ethnic-gender subgroup. Inverse relations were noted between FMD and both BMI and height in the overall MA cohort (r = -0.205, p < 0.05 and r = -0.223, p < 0.05, respectively), and also between FMD and height and weight in the overall NHW cohort (r = -0.257, p < 0.01, and r = -0.216, p < 0.05, respectively). In addition, in MA men, FMD was inversely related to all body size measures, whereas in NHW men, FMD was directly related to diastolic BP and HDL-cholesterol.

Of additional interest, in MA men, an inverse relationship was noted between urinary albumin and FMD (r = -0.26, p < 0.05). In contrast, no significant relationship was found between FMD and urinary albumin in MA women, or in NHW men or women. Furthermore, in women in both ethnic groups, no significant correlation was observed between any of the continuous or categorical variables and FMD-except for an inverse relation between Alb:Cr ratio and FMD in MA women.

Study participants were further subgrouped based on ethnicity, gender and FMD values (FMD ≥ 7% vs FMD < 7%). LV mass in the overall MA cohort with FMD ≥ 7% was significantly lower than in the overall NHW cohort with FMD ≥ 7% (p = 0.03), and lower in MA with FMD < 7% compared with NHW participants with FMD < 7%. Urinary albumin values were lower in the overall MA cohort than in the overall NHW cohort and in MA women with FMD% ≥ 7 than in MA women with FMD < 7% (0.61 ± 0.7 vs 0.95 ± 0.8 mg/dl and 0.62 ± 0.5 vs 1.1 ± 1.1 mg/dl, p < 0.006 and 0.05, respectively). In contrast, there was no significant relation between urinary albumin levels and FMD < 7 vs ≥ 7% in the NHW cohort (0.76 ± 1.2 vs 0.69 ± 0.5 mg/dl).

**Figure 1:**
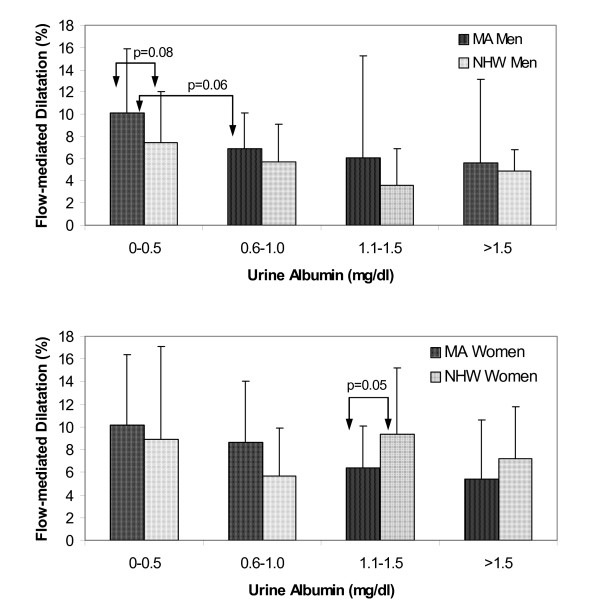
Flow-mediated dilatation and urine albumin levels in Mexican-American and non-Hispanic white men (top panel) and women (bottom panel).

The relationship between FMD and urinary albumin is displayed in Figure 1 for MA versus NHW men and for MA versus NHW women. Overall, FMD in MA and NHW men and women decreased as urinary albumin levels increased. Among women with urinary albumin levels of 1.1–1.5 mg/dl, FMD was significantly higher in MA than in NHW women (p < 0.05) (Figure 1). In MA men with urinary albumin levels of 0–0.5 mg/dl, FMD trended higher than in NHW men with the same albumin levels (p < 0.08). Meanwhile, in MA men with urinary albumin levels of 0.0–0.5 mg/dl, FMD was nearly significantly higher than in MA men with urinary levels of 0.6–1.0 mg/dl (p < 0.06). In NHW men with urinary albumin levels of 0–0.5 mg/dl, FMD trended higher than in NHW men with urinary albumin levels of 1.1–1.5 mg/dl (p = 0.054).

### Multiple linear regression

In MA men, BMI, systolic BP and urine albumin were predictor variables and inversely related to FMD. After age, brachial artery baseline diameter, BMI, systolic BP, LDL-cholesterol, HDL-cholesterol, triglycerides, glucose, and current smoking were forced into the multiple linear regression model, the *inverse association of FMD and urinary albumin persisted *(Table 2). In MA women, BMI, urine albumin and Alb:Cr ratio (β = -0.140, -28.57, and β = -0.211, respectively) were the most important predictor variables, and inversely related to FMD (all p < 0.05). After forcing the other variables listed above into the model, an inverse association of FMD to Alb:Cr ratio persisted in MA women (p < 0.05). In NHW men, only BMI and HDL-cholesterol were positive predictors of FMD, while in NHW women, only age and BMI were predictor variables, and inversely related, to FMD. In both NHW men and women participants, urinary albumin and Alb:Cr ratio were not associated with FMD after entering the above variables (Table 2).

**Table 2:** Multiple linear regression of CHD risk factors and urinary albumin versus flow-mediated dilatation in Mexican-American and non-Hispanic white men and women

	Flow-Mediated Dilatation (%)		Flow-Mediated Dilatation (%)	
	Standardized β-Coefficient		Standardized β-Coefficient	
Variable	MA men	MA women	NHW men	NHW women
Constant	-4.848	23.7	-22.240	4.3
Age (years)	0.042	-0.090	-0.045	-0.230*
BA Baseline Diameter	0.053	0.069	0.081	0.074
BMI	-15.29*	-0.140*	0.539*	-0.241*
Systolic BP (mmHg)	-.311*	0.013	0.039	0.074
LDL-Cholesterol (mg/dl)	0.002	-0.077	-0.009	-0.001
HDL-Cholesterol (mg/dl)	-0.084	-0.014	0.154*	0.081
Triglycerides (mg/dl)	-0.006	0.002	0.019	0.012
Serum Glucose (mg/dl)	-0.009	0.033	-0.018	0.026
Current Smoking (1 = yes)	-0.031	0.001	-0.002	0.112
Urine Albumin (mg/dl)	-26.95**	-28.57*	3.425	-15.973
Alb: Cr Ratio	-0.118	-0.211*	-0.046	0.147

*p < 0.05, ** p < 0.01, LDL, low density lipoprotein; HDL, high density lipoprotein; Alb:Cr ratio, Albumin:creatinine ratio

### ANCOVA analyses

FMD was significantly higher in MA compared with NHW individuals. Of interest, after adjustment for age alone, age plus CVD risk factors and brachial artery baseline diameter or age, CVD risk factors plus albumin or plus Alb:Cr ratio, FMD was not found to be significantly different between MA and NHW men or between MA and NHW women. After adjusting for age, CVD risk factors plus urinary albumin, FMD was significantly higher only in MA men compared to NHW men (7.8 ± 6.4% vs. 5.6 ± 4.5%, p < 0.04). Alb:Cr ratio was not significantly associated with FMD in either cohort.

FMD has been reported to be useful to assess long-term CVD risk in high-risk and lower-risk populations, and to predict short-term postoperative CVD event risk in a high-risk population [14,15]. Endothelial function in normal healthy adults is influenced by variables such as race [11,14], age [13,14,16], and prandial state [17]. In healthy people, endothelial function, as measured by FMD, has been reported to be in the range of 7 to 10% (mean) [11-14], but in patients with CVD, FMD is impaired or absent, with FMD values ranging from 0 to 5%.

In our study, the MA cohort demonstrated significantly higher FMD in comparison with the NHW cohort (9.1 ± 7.3% vs 7.1 ± 6.3%, p < 0.04). These findings are of interest in view of a report from Bild and co-workers that the prevalence of CAC, measured from computed tomographic (CT) scanning, was lower in a Hispanic (55.6%) compared to a non-Hispanic Caucasian (70.4%) cohort^5^. Ethnic differences in endothelium-dependent FMD have also been observed in Chinese subjects, compared to white subjects from Australia [18], and in Indian Asians in the United Kingdom compared with European whites [16].

Our findings of increased FMD in asymptomatic MA versus NHW, with no significant ethnic differences in baseline brachial artery diameter, may be due to a number of factors – e.g., an increase in eNOS activity in the MA versus the NHW cohort, the presence of cohort differences in other non-evaluated CHD risk factor(s) or biomarkers which also modulate endothelial function, other environmental or dietary factors, or perhaps genetic differences. A consistent, but non-significant increase in % FMD in both MA and NHW women, versus MA and NHW men, may be due to smaller BA diameters in women than men (Table 1).

FMD has been shown previously to be decreased in obese individuals [19]. In our study, FMD was lower in the asymptomatic MA cohort and was inversely associated with BMI. In contrast, there was no such association in NHW. This inverse relation between BMI and FMD in Hispanics is similar to a previous report [18].

Impaired vasodilatation of vascular endothelium (which predates atherosclerotic deposition) has been observed in apparently healthy patients with risk factors for CHD, including type 2 diabetes mellitus, but normoalbuminuria [20,21]. It has been suggested that endothelial dysfunction may be an earlier predictor than urinary albumin of the development of CHD and cardiovascular risk [21,22]. Nonetheless, higher urinary albumin values, even at levels below clinical microalbuminuria, are generally associated with several measures of subclinical CVD in adults without established CVD [20,23,24].

In our MA, but not our NHW cohort, urinary albumin levels were consistently higher in subjects with FMD < 7% versus those with FMD ≥ 7%. Urinary albumin levels were the only predictor (inverse) in multivariate analyses of FMD in MA men, while urinary Alb:Cr ratio was an inverse predictor of FMD in MA women. Of interest, in patients with persistent microalbuminuria, FMD has been reported to be significantly less in Caucasians than in African-Americans [17].

### Limitations

The sample size of our pilot study was relatively small, and a substantial proportion of the variability of brachial FMD in our cohort remains unexplained by standard CVD risk factors. A larger sample size might have helped to better characterize the contribution of race/ethnicity to FMD variability. The lack of an automated system for FMD analysis may be an added limitation in the study.

## Conclusion

To our knowledge, this is the first study to analyze, in *asymptomatic *adults, the relation of MA and NHW ethnicity to FMD and urinary albumin levels. Overall, our MA cohort demonstrated higher FMD compared to the NHW cohort. Our results are consistent with growing evidence that risk factors contribute to the development of CVD, at least in part, by impairing endothelial function. Longitudinal follow-up of MA and NHW cohorts should help determine whether endothelial dysfunction, as measured by FMD, and urinary albumin provide additional value in predicting CVD outcomes in additional to traditional risk factors.

## Competing interests

The authors declare that they have no competing interests.

## Authors' contributions

JMD have made substantial contributions to conception and design of the study, interpretation of data and writing of the manuscript and have given final approval of the version to be published. ZA performed the statistical analysis and drafting and revising the manuscript. NDW participated in the design of the study and revising the manuscript critically for important intellectual content. SKS instrumental in the recruitment of study participants and in acquisition of the data. RLB carried out the echocardiography and flow-mediated dilatation studies. MAS participated in the study design and coordination and helped to draft the manuscript. HAP participated in the study design and coordination and helped to draft the manuscript. All authors read and approved the final manuscript.

## Acknowledgements

This research was supported by a Grant-in-Aid from the American Heart Association, National Association (99505-47N) to Dr. Gardin and by funding from St. John Guild Cardiovascular Research Endowment.
